# The Nefashot Initiative’s Journey: Transforming from Local Activism into a Diverse Community Promoting Mental Health through Arts and Culture

**DOI:** 10.1192/j.eurpsy.2024.1408

**Published:** 2024-08-27

**Authors:** S. Regev, R. Diller

**Affiliations:** ^1^Department of Health Policy and Management, Ben-Gurion University of the Negev, Be’er Sheva; ^2^Nefashot, Jerusalem, Israel

## Abstract

**Introduction:**

*Nefashot*, meaning ‘Souls’ and ‘People’ in Hebrew, emerged from a local group of impassioned activists. Our core mission is to promote mental health awareness through cultural and artistic expressions, bridging gaps in open and inclusive conversations.

**Objectives:**

In our early years, our primary goal was to infuse MH discussions into public spaces through art. However, as our community has evolved, so too have our objectives. Today, we recognize the profound impact of these connections and discussions, both within and beyond our dynamic community. We’ve come to understand that belonging to this community is, in itself, a catalyst for change. This shift in perspective has allowed us to fully embrace the transformative potential of community engagement and direct our activities.

**Methods:**

At the heart of our approach is the nurturing of a profound sense of belonging within our diverse community. We achieve this through two vital activities: (1) Ongoing Community Communication: Within our dynamic community, which includes individuals dealing with mental health challenges, professionals, family members, friends, and allies, communication is paramount to our unity. We maintain an open and continuous dialogue through a dedicated WhatsApp group. This platform facilitates connection, sharing of experiences, and mutual support, strengthening the bonds that unite us in our shared mission. (2) Community-Driven Event Production: Beyond our annual ‘Osim Nefashot’ week, held around World Mental Health Day, we seize opportunities throughout the year to organize events or collaborate with larger events like International Women’s Day in March or Book Week in June. Encouraging active participation in event planning and execution not only amplifies the voices of our members but also deepens their sense of belonging within our ever-evolving community.

**Results:**

Our hallmark is a sustainable process, welcoming new creators, forging connections, and expanding our influence while retaining core members. This renewal and continuity enable us to reach new audiences and expand mental health awareness through art and culture. Our growth is showcased, with 90 events organized last year.

**Conclusions:**

Nefashot’s transformation from activists to a diverse community is an ongoing journey requiring commitment and deliberate steps. Our allocated resources and activities ensure each participant, from creators to venues to attendees, plays a vital role in advancing mental health awareness. We remain dedicated to nurturing inclusivity and promoting mental well-being through art and culture.

**Disclosure of Interest:**

None Declared

